# Spectral prediction of anthocyanin concentration in *Populus pruinosa* leaves based on vegetation index

**DOI:** 10.3389/fpls.2026.1787326

**Published:** 2026-03-23

**Authors:** Huixia Li, Jiaqiang Wang, Wenhao Xia, Ben Wang, Shaoying Sun, Chongfa Cai

**Affiliations:** 1 College of Life Science and Technology, Tarim University, Alar, China; 2 College of Agriculture, Tarim University, Alar, China; 3 Key Laboratory of Genetic Improvement and Efficient Production for Specialty Crops in Arid Southern Xinjiang Production and Construction Corps, Tarim University, Alar, China; 4 Research Center of Oasis Agricultural Resources and Environment in Southern Xinjiang, Tarim University, Alar, China; 5 College of Resource and Environment, Huazhong Agricultural University, Wuhan, China

**Keywords:** anthocyanin, hyperspectral reflectance, machine learning, *Populus pruinosa*, vegetation index

## Abstract

**Introduction:**

*Populus pruinosa* is the key foundation tree species in desert riparian forests in arid areas of northwestern China. Timely and accurate monitoring of the physiological status of *P. pruinosa* is crucial for restoring the damaged ecosystem. Anthocyanins are one of the important physiological indicators that reflect the environmental adaptability of *P. pruinosa* under stress. Existing studies have extensively applied hyperspectral technology for the quantitative prediction of crop leaf pigments. However, research on hyperspectral prediction of anthocyanin concentration in woody halophytes is still lacking, particularly in the integration of spectral preprocessing, species-specific vegetation index construction, and machine learning modeling.

**Methods:**

In this study, the hyperspectral technology was used to estimate the anthocyanin concentration of *P. pruinosa* leaves collected in five months (June - October) under five groundwater depth conditions (0-2, 2-4, 4-6, 6-8, and 8-10 m). Based on first-order (FD) and second-order (SD) derivative processing, competitive adaptive reweighted sampling (CARS), https://xueshu.baidu.com/usercenter/paper/show?paperid=bea4d6371f19161f21aac22941cc4408&site=xueshu_se shuffled frog leaping algorithm (SFLA), and recursive feature elimination with cross-validation (RFECV) were used to extract spectral features of *P. pruinosa* leaves to construct the anthocyanin reflectance index, composite index, difference vegetation index, and normalized anthocyanin reflectance index. After that, the top 10 sets of data with high correlation with anthocyanin concentration were selected from each vegetation index to form a total data set (40 sets in total) for modeling. Twelve models were constructed using support vector machine (SVM) and one-dimensional convolutional neural network (1D-CNN) methods.

**Results:**

The FD and SD derivative transformations of the spectral reflectance significantly enhanced the correlation with anthocyanin concentration. The feature extraction methods SFLA and RFECV were superior in extracting the bands highly related to anthocyanin concentration, and the vegetation indices constructed based on these two methods had a high correlation with anthocyanin concentration in the red and near-infrared regions. The optimal prediction model was FD-SFLA-SVM (R^2^ = 0.852, RMSE = 86.851 mg m^-2^, RPD = 2.596).

**Discussion:**

Unlike existing vegetation index-based studies, the research develops a systematic approach to construct vegetation indices and models for estimating the anthocyanin concentration in the woody halophyte *P. pruinose* in deserts. The research will provide technical support for non-destructive monitoring of the physiological status of *P. pruinosa*, and also contribute to the restoration of desert riparian ecosystems.

## 1 Introduction

*Populus pruinosa*, as the only foundation tree species in desert riparian forests in Xinjiang, China, plays a crucial role in maintaining the stability of fragile ecosystems. Its health status directly reflects the resilience of desert riparian ecosystems under the continuous intensification of climate change and environmental stress (Yu et al., 2020). Anthocyanins are the most widely found secondary metabolites in plants (Zhou et al., 2024). Anthocyanins not only affect the color of plant leaves but also are closely related to plant physiological conditions (Miao et al., 2024). They can enhance plant antioxidant, stress resistance, and disease resistance (Zhao et al., 2023; Ahammed and Yang, 2022). As plant osmolytes, anthocyanins protect plants from various abiotic stresses through osmotic regulation (Altangerel et al., 2017), making them key physiological indicators for assessing plant adaptation to the environment. Therefore, anthocyanin concentration can be used to explore the growth status and stress tolerance of *P. pruinosa* under high salinity and drought conditions. Hyperspectral technology, with fast, non-destructive, and high-precision characteristics (Thoreau et al., 2024), is an important means for precision agriculture and vegetation growth monitoring. Previous studies have extensively applied hyperspectral technology for quantitatively predicting the physiological parameters of crops such as cotton and rice (Li et al., 2024; Cao et al., 2020). However, research on hyperspectral technology-based prediction of anthocyanin concentration in woody halophytes in the arid areas of northwest China remains limited. Notably, vegetation indices constructed from crop data cannot be directly applied to tree species because of their vastly different structural and physiological characteristics. Currently, there are few studies that construct specific vegetation indices based on the spectral response characteristics of *Populus pruinosa* anthocyanins, which limits the quantitative prediction accuracy and stability.

The accuracy of hyperspectral technology-based predictions can be enhanced through various methods. Hyperspectral data preprocessing is the key to constructing high-precision prediction models (Li et al., 2024). For example, Moura-Bueno et al. (2019) reported that the derivative transformation after Savitzky–Golay smoothing of the raw spectrum significantly enhanced model performance. Liu et al. (2024) enhanced the prediction accuracy of anthocyanin concentration in maize and soybean leaves using first-order derivative (FD) and standard normal variate (SNV) preprocessing of the hyperspectral data. In addition, vegetation indices constructed based on the spectral features of pigments are widely used for the quantitative prediction of anthocyanin and chlorophyll concentrations in crops (Kim and van Iersel, 2023; Zhang et al., 2021). Gao et al. (2024) developed a method for predicting the chlorophyll content of soybean leaves using the Back Propagation Neural Network combined with vegetation indices. Zhang et al. (2023) enhanced the prediction accuracy and stability of anthocyanin concentration in apple leaves by constructing spectral indices significantly correlated with anthocyanin concentration and applying the Sparrow Search Algorithm-Random Forest (SSA-RF). These studies provide references for developing anthocyanin-specific vegetation indices for *Populus pruinosa*.

**Figure 1:**
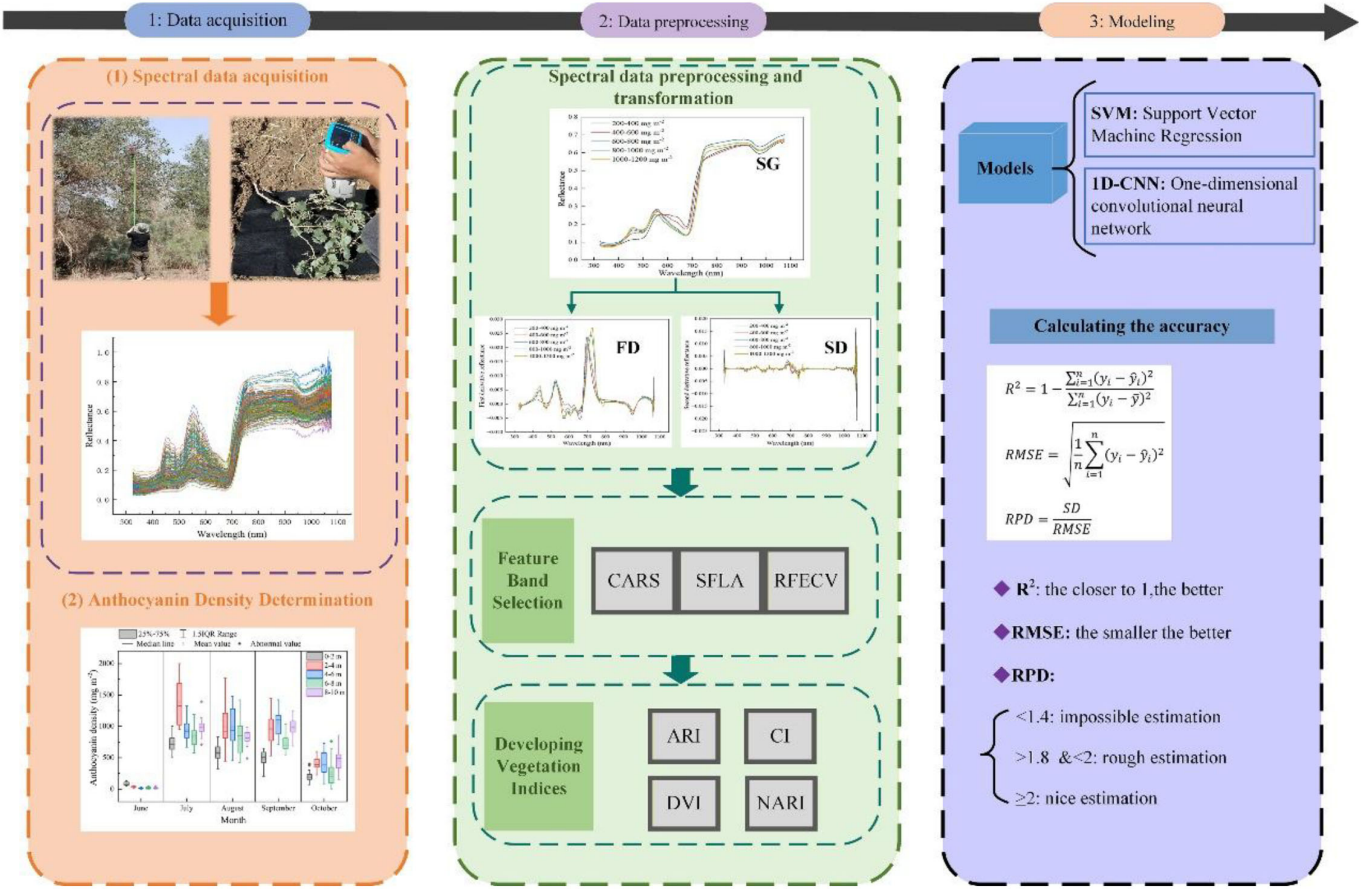
The framework of this study.

Currently, progress has been made in the study of plant anthocyanins using spectroscopic techniques. However, estimating *P. pruinosa* anthocyanin under drought stress by integrating spectral preprocessing optimization, vegetation index construction, and machine learning modeling has not been fully explored. Therefore, this study extracted the spectral features of *P. pruinosa* anthocyanins after spectral preprocessing and constructed vegetation indices, followed by machine learning modeling. The proposed method was expected to accurately and stably reveal the changes in leaf anthocyanin of *P. pruinosa* under drought stress (**Figure 1**). The study results will provide technical support for non-destructive monitoring of the physiological status of *P. pruinosa* and contribute to the restoration of desert riparian ecosystems.

## 2 Materials and methods

### 2.1 Experimental area

**Figure 2:**
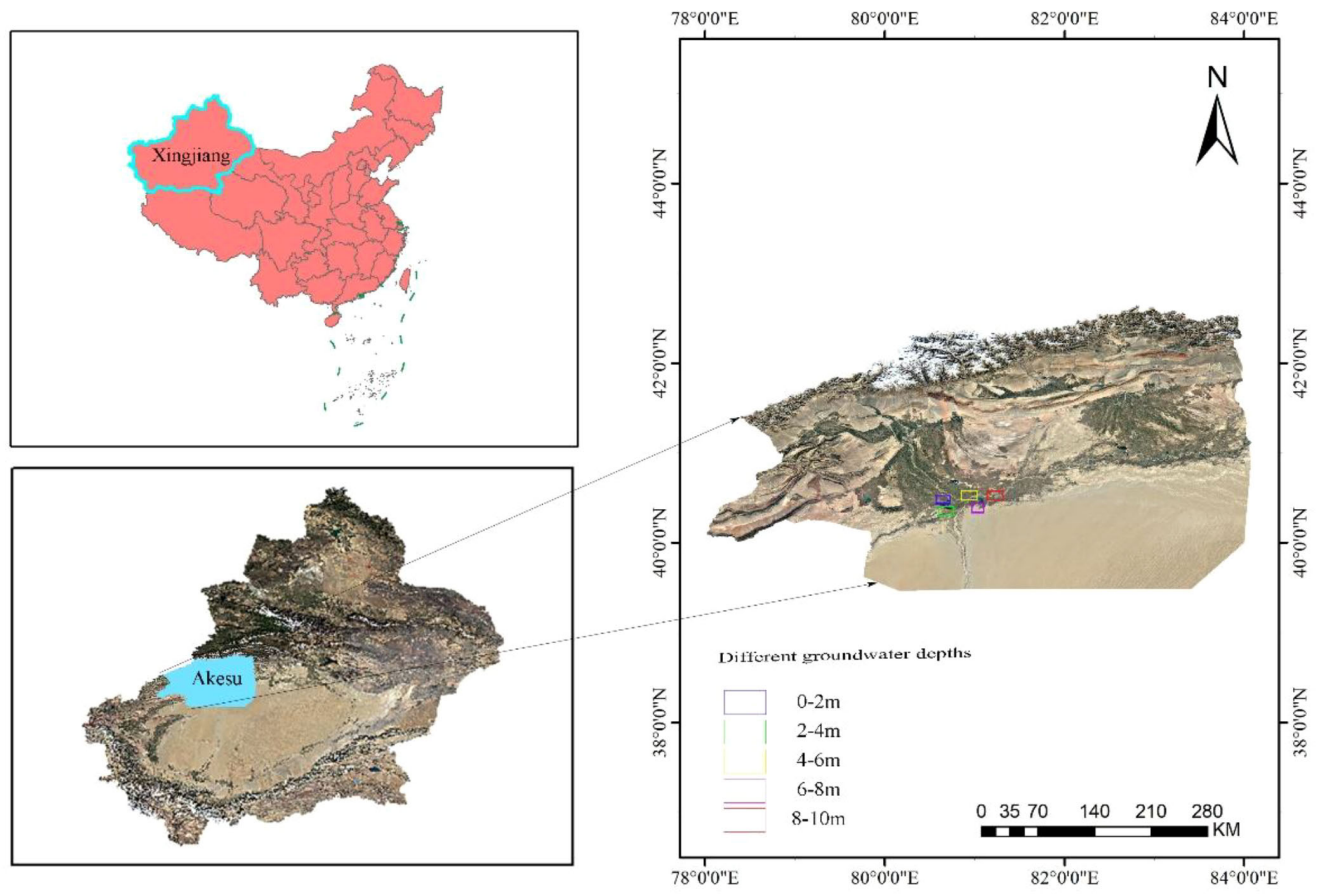
The study areas.

The research area is located in the *P. pruinosa* Forest Reserve in Fengshou Sanchang in Awati County (80°19’54.71”-80°24’13.10”E, 40°15’53.72”-40°18’23.13”N) (**Figure 2**). The *P. pruinosa* Forest Reserve is situated in the lower reaches of the Yarkand River in the upstream source area of the Tarim River, with a temperate continental climate (low rainfall and strong evaporation). Its water resource is mainly glacier meltwater, and the groundwater depth is less affected by rainfall. The source area of the mainstream of the Tarim River is located at the confluence of the Aksu River, Hotan River, and Yarkand River. This area has a temperate continental climate, with frequent drought and little rainfall. It is affected by floods from July to September every year. In this study, five *P. pruinosa* forests with different groundwater depths (0-2, 2-4, 4-6, 6-8, and 8–10 m) were selected within the study areas. The areas with groundwater depths of 0–2 and 2–4 m are located at the source of the Tarim River mainstream (groundwater depth was measured using drilling and soil excavating), while the areas with groundwater depths of 4-6, 6-8, and 8–10 m are located in the *P. pruinosa* Forest Reserve in Fengshou Sanchang in Awat County (groundwater depth was measured by drilling).

### 2.2 Data acquisition

#### 2.2.1 Spectral data acquisition

The FieldSpec portable spectrometer (HandHeld2, ASD Company, USA) was used to measure the spectral reflectance of *P. pruinosa* leaves in 325-1,075 nm from 12:00 to 16:00 on a cloudless day. During the measurement, the probe was vertically downward and kept at a distance of 10 cm from the sample. At the same time, a black plate was used as the background. Whiteboard correction was performed before measurement to address issues such as drift and decreased sensitivity. During measurement (June-October 2023), whiteboard correction was performed every 10 minutes. Samples were collected in consecutive 3-day periods in the middle of each month. In each month, twenty *P. pruinosa* trees were randomly selected from each *P. pruinosa* forest (five in total). Twenty old leaves of each tree were selected for measurement, and the average value was calculated. A total of 500 leaf samples were collected, and their hyperspectral data were acquired.

#### 2.2.2 Measurement of anthocyanin concentration

In this study, the anthocyanin concentration per unit leaf area (hereafter referred to as anthocyanin concentration) was measured to investigate the variation characteristics of anthocyanin concentration in *P. pruinosa* leaves under different groundwater depth conditions in different months. Outlier removal and normality analysis were performed on anthocyanin concentration data.

After acquiring the spectral data of each sample, the fresh leaf samples were weighed, followed by the measurement of leaf area using the LI-3000C leaf area meter (Li-COR, Lincoln, NE, USA). Finally, the specific leaf weight (SLW) was calculated (Equation 1). According to the method of Sims and Gamon (2003), anthocyanins were extracted from the leaf samples using acetone and Tris buffer (V: V = 8: 2), followed by colorimetry using a UV-visible spectrometer (model: UV5600) to obtain the absorbance at 470 (A_470_), 537 (A_537_), 647 (A_647_), and 663 (A_663_) nm. Then, the amount of anthocyanins in a unit volume of solution was calculated. Finally, the leaf anthocyanin concentration per unit area was calculated.

(1) $$SLW(m^{2}g^{-1}\mu molml^{-1}mgm^{-2})=S/W_{F}$$

(2) $$C_{Ant\;}=0.08173A_{537}-0.00697A_{647}-0.002228A_{663}$$

(3) $$Ant\left(mgm^{-2}\right)=C_{Ant}\cdot V\cdot M_{Ant}/\left(1000\cdot W_{F}\cdot SLW\right)$$

where *S* is leaf area, *W_F_* is fresh leaf weight, *C_Ant_* is the amount of anthocyanins in a unit volume of solution (Equation 2), *A* is the absorbance, *Ant* is leaf anthocyanin concentration per unit area (Equation 3), *V* is the volume for colorimetry, and *M* is molecular mass.

### 2.3 Data preprocessing

#### 2.3.1 Spectral data preprocessing and transformation

This study preprocessed spectral data in different ways with the aim of reducing data redundancy, removing high-frequency noise, and minimizing mutual interference between noises. Python 3.8 was used to resample the acquired spectral data at an interval of 2.5 nm to maintain consistent resolution across different wavelengths. Savitzky-Golay smoothing was then used to suppress noise in the resampled spectral data to obtain the raw spectrum. After that, first-order and second-order derivative transformations were performed on the raw spectrum.

#### 2.3.2 Spectral feature extraction

Three methods were used for spectral feature extraction:

Competitive adaptive reweighted sampling (CARS) is a widely used wavelength selection algorithm based on PLS (partial least squares) regression coefficients (Yang et al., 2025). It randomly selects a portion of samples from the calibration set through Monte Carlo simulation (MCS) to construct a PLS model. Then, adaptive reweighted sampling is performed to competitively extract the initial feature variables. After that, the final feature variables are selected from the wavelength set based on the root mean square error of cross-validation (RMSECV).

The shuffled frog leaping algorithm (SFLA) is a variable optimization algorithm. This method models by iterating with a small number of variables. It calculates the selection probability of each variable, drawing inspiration from the foraging behavior of frogs. The selection probability reflects the importance of the variable. After sorting by selection probability and continuously removing variables with low importance, the RMSECV is obtained by modeling. The subset of variables with the smallest RMSECV is selected as the optimal spectral feature set (Li et al., 2012).

Recursive feature elimination with cross-validation (RFECV) is a feature selection method that combines recursive feature elimination (RFE) and cross-validation (CV). This method evaluates the performance of the model after removing each feature through cross-validation. It can reduce the dimensionality of features and improve the generalization ability of the model while ensuring prediction accuracy. The core advantage lies in repeatedly constructing models to select the most influential feature subset for the target task. This optimizes the classification performance of the model, reduces computational overhead and training time, improves algorithm efficiency and reliability, and reduces the risk of overfitting (Sharma and Singh, 2024).

#### 2.3.3 Construction of anthocyanin-related vegetation indices

Vegetation index is a mathematical combination based on the reflectance of two or more spectral bands (Huete et al., 2002). It is widely used to monitor vegetation status and dynamic changes. The essence of the vegetation index is to perform normalization and other operations on the spectral reflectance. The use of vegetation indices can significantly enhance the model accuracy. Multi-band combination can reduce the influence of sensors and background on targets, and achieve data dimensionality reduction (Inoue et al., 2012). In this study, while retaining the band order (i.e., Bi, Bj ≠ Bj, Bi), all possible two-band combinations were generated. Then, four vegetation indices highly correlated with anthocyanin concentration were constructed based on a preset mathematical form, namely, anthocyanin reflectance index (ARI), composite index (CI), difference vegetation index (DVI), and normalized anthocyanin reflectance index (NARI) (**Table 1**). Origin 2024 (OriginLab, MA, USA) was used to analyze the correlation between vegetation indices and anthocyanin concentration. The top 10 vegetation indices with high correlation with anthocyanin concentration were selected. Finally, 40 sets of feature subsets were obtained to construct the model.

**Table 1:** Optimized vegetation indices.

Vegetation index	Formula	Reference
Anthocyanin reflectance index (ARI)	$1/R_{i}-1/R_{j}$	Gitelson et al., 2001
Composite index (CI)	$\left(R_{i}-R_{j}\right)/R_{j}$	Zhang et al., 2021
Difference vegetation index (DVI)	$R_{i}-R_{j}$	Lin et al., 2021
Normalized anthocyanin reflectance index (NARI)	$\left(1/R_{i}-1/R_{j}\right)/\left(1/R_{i}+1/R_{j}\right)$	Bayle et al., 2019

R_i_ and R_j_ represent the spectral reflectance of any two bands highly related to anthocyanin concentration, and i and j represent any two bands highly related to anthocyanin concentration.

### 2.4 Modeling

#### 2.4.1 Modeling methods

In this study, MATLAB software (R2024a, MathWorks, USA) was used to process the spectral reflectance and anthocyanin concentration data of *P. pruinosa* leaves. The dataset consisted of 303 samples (after outlier removal). Using stratified random sampling, the anthocyanin concentrations of the samples (0.9–1,477.3 mg m^−2^) were arranged in ascending order and evenly divided into five intervals. Within each interval, samples were independently randomly selected at a 7: 3 ratio, forming the modeling (212 samples) and validation (91 samples) sets, respectively. The mean, standard deviation, and value range of the two sets were highly consistent, ensuring the representativeness of the data. The combined vegetation index (40 feature subsets) calculated using first-order and second-order derivative spectral reflectance was used to construct a relationship with the measured anthocyanin concentration. Finally, the anthocyanin concentration of *P. pruinosa* leaves was predicted.

#### 2.4.2 Support vector machine

The SVM is developed based on statistical learning theory. Its core is to use kernel functions to implicitly map data to a high-dimensional feature space, ultimately constructing the optimal hyperplane in the high-dimensional space. The SVM has excellent generalization ability. Its structure is related to the selection of the kernel function. The kernel function varies depending on different experimental samples (Rezvani et al., 2019). In this study, the SVM algorithm used a radial basis function as the kernel function.

#### 2.4.3 One-dimensional convolutional neural network

The 1D-CNN is a deep learning model that extracts features from hyperspectral data through one-dimensional convolution operations. The 1D-CNN has strong nonlinear modeling capabilities and can capture local patterns and inter-band relationships in spectral data. Through multiple convolutional layers, the model can automatically learn the complex nonlinear relationship between vegetation index and anthocyanin concentration (LeCun et al., 2015).

### 2.5 Accuracy verification

The coefficient of determination (R^2^), root mean square error (RMSE), and relative prediction deviation (RPD) were selected to test the accuracy of the model. The closer the R^2^ value is to 1, the better the model fits (Equation 4). RMSE represents the deviation between the predicted and measured values (Equation 5). The smaller the value, the better the model fits. RPD represents the stability of the model (Equation 6), and RPD ≥ 1.4 is the threshold. When 1.8< RPD< 2, the model prediction performance is good. When RPD ≥ 2, the model is very good and can be used for high-precision prediction tasks (Rossel et al., 2006).

(4) $$R^{2}=1-\frac{\sum_{i=1}^{n}{\left(y_{i}-{\hat{y}}_{i}\right)}^{2}}{\sum_{i=1}^{n}{\left(y_{i}-\bar{y}\right)}^{2}}$$

(5) $$RMSE=\sqrt{\frac{1}{n}\sum_{i=1}^{n}{\left(y_{i}-{\hat{y}}_{i}\right)}^{2}}$$

(6) $$RPD=\frac{SD}{RMSE}$$

where n is the number of samples, *y_i_* and ${\hat{y}}_{i}$ represent the measured and predicted values of the i-th sample, $\bar{y}$ is the average of the measured values, and SD is the standard deviation.

## 3 Results

### 3.1 Characteristics of anthocyanin concentration changes in *P. pruinosa* leaves

**Figure 3:**
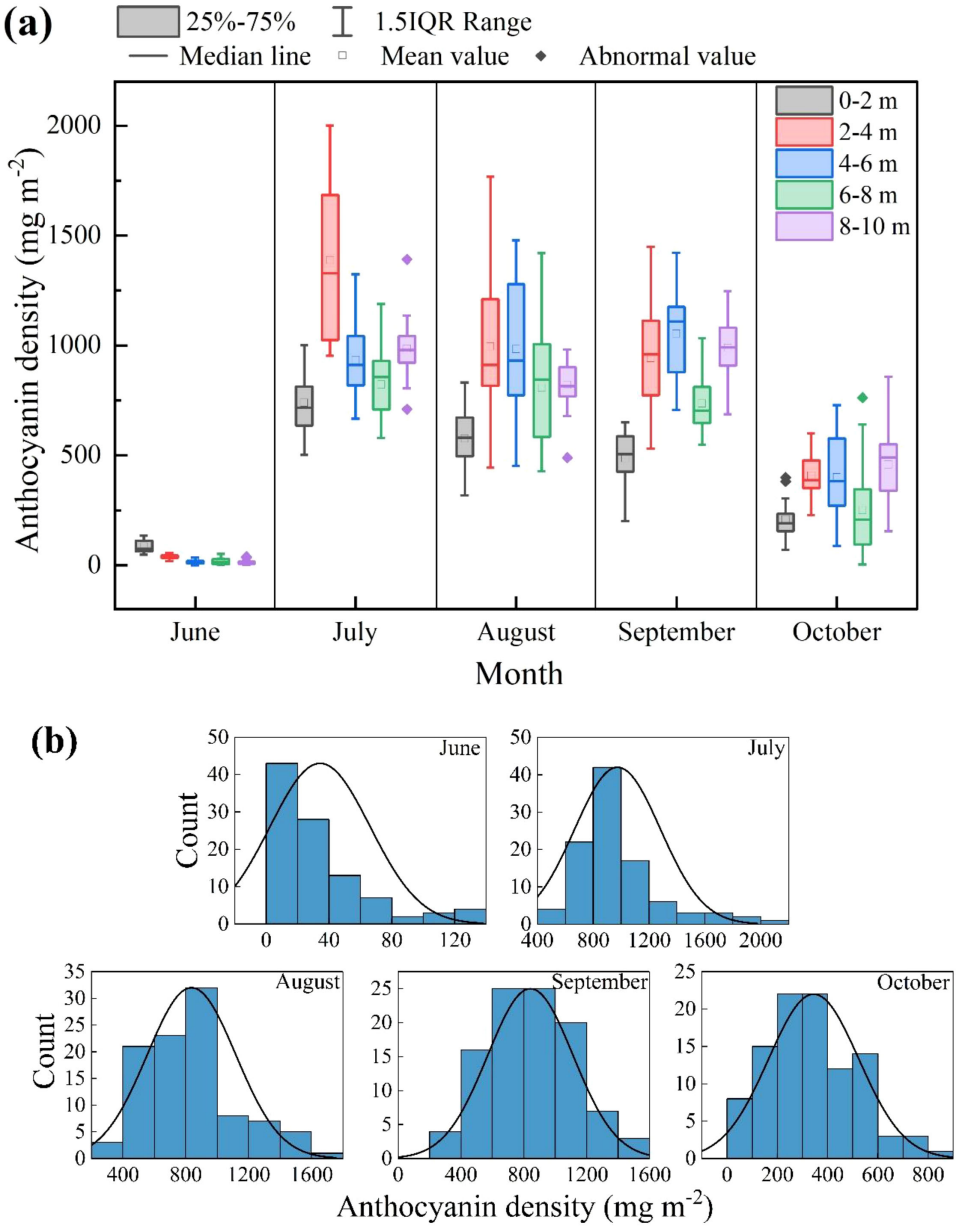
Changes in anthocyanin concentration in *P. pruinosa* leaves in different months under different groundwater depth conditions. **(a)** Changes of anthocyanin concentration in *P. pruinosa* leaves; **(b)** Distribution of anthocyanin concentration in different months.

The anthocyanin concentration of *P. pruinosa* leaves under different groundwater depth conditions showed a trend of first increasing and then decreasing over time, showing significant seasonal differences (**Figure 3a**). The anthocyanin concentration in each month ranged from 0.9 to 1,477.3 mg m^-2^. The selected 5 months can characterize the changing trend of *P. pruinosa* growth (**Figure 3b**).

Among the five months, June had the lowest anthocyanin concentration, and the anthocyanin concentration varied little across different groundwater depths. The median anthocyanin concentration of *P. pruinosa* leaves was only 13.9 mg m^-2^ under the 4–6 m groundwater depth condition (4–6 m group). In July-August, the anthocyanin concentration showed a significantly increasing trend over time. Among them, the anthocyanin concentration of the 2–4 and 4–6 m groups was higher than that of other months, with a median higher than 909.6 mg m^-2^, indicating an accelerated accumulation of anthocyanins in leaves during the summer season. By October, anthocyanin concentration showed a significantly decreasing trend, and the range of variation narrowed. The leaf anthocyanin concentration of the 0–2 m group was generally low in each month. In the late stage of *P. pruinosa* growth (September and October), the leaf anthocyanin concentration of the 8–10 m group was significantly higher than that of other groups.

### 3.2 Spectral characteristics of *P. pruinosa* leaves

**Figure 4:**
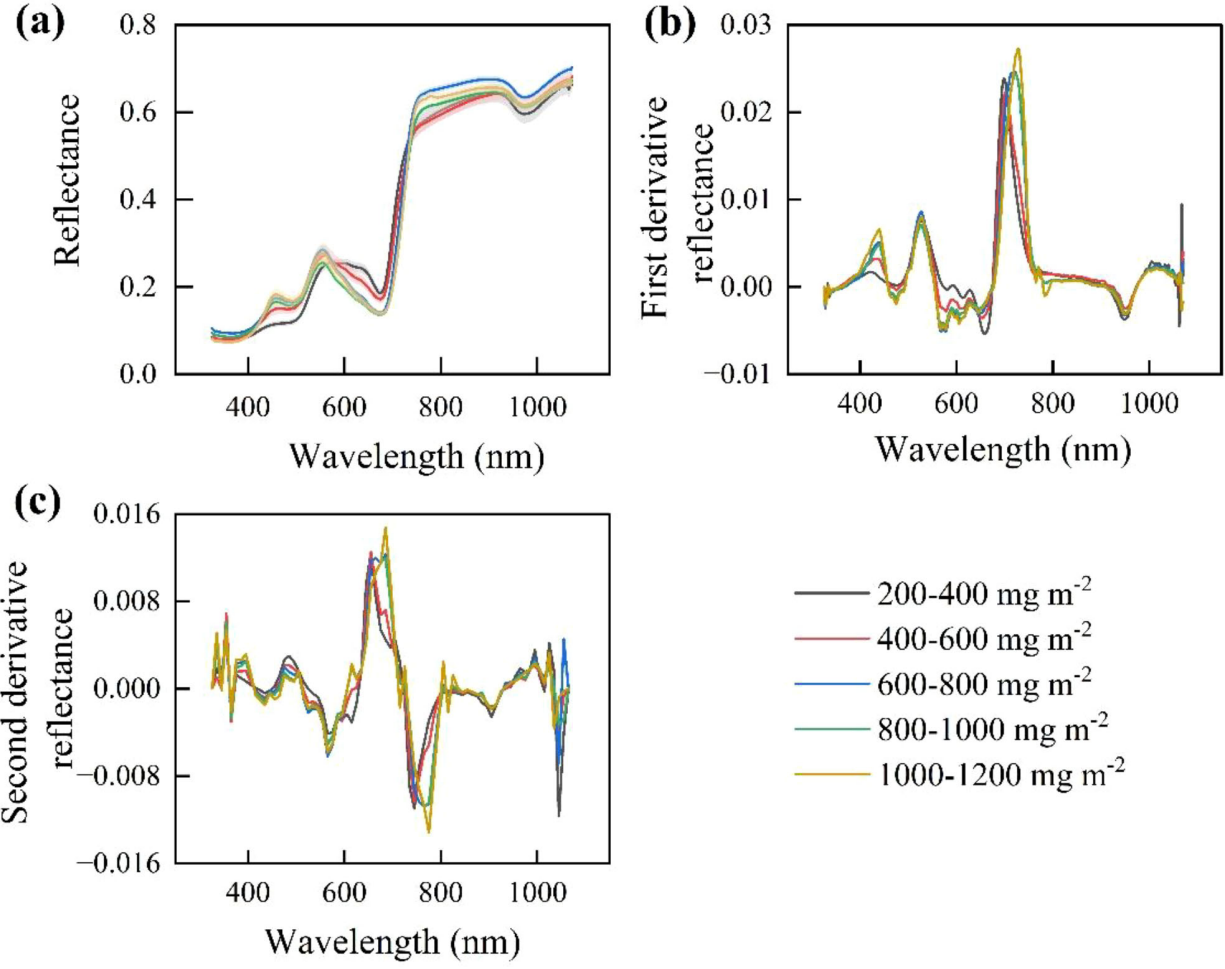
Spectral reflectance variation for different anthocyanin concentration intervals of *P. pruinosa* leaves. **(a)** Raw spectral features. **(b)** First-derivative spectral features. **(c)** Second-derivative spectral features. The anthocyanin concentrations were classified into five intervals: 200-400, 400-600, 600-800, 800-1000, and 1000–1200 mg m^-2^.

The spectral reflectance of *P. pruinosa* leaves exhibited a significant band-specific response to the variation of anthocyanin concentration. The bands with significant differences in reflectance (*p* < 0.05) were 415-510, 580-680, and 750–920 nm, among which the bands with regular features were concentrated in 410–700 nm (**Figure 4a**). In the blue region (415–510 nm), the spectral reflectance increased with the increase of anthocyanin concentration. In the red region (580–680 nm), there was a negative correlation between reflectance and anthocyanin concentration. However, in the near-infrared region (750–920 nm), the reflectance range was 0.58 - 0.67, and the spectral reflectance did not show a significant change pattern. Within this range, the reflectance was highest (0.66) when the anthocyanin concentration was in the range of 600–800 mg m^-2^, while the reflectance outside of this range was all below 0.64.

The first-order and second-order derivative spectral curves had more peaks than the raw spectrum, with more prominent features (**Figures 4b, c**). There were significant differences in spectral reflectance in the visible region, which was consistent with the variation of the raw spectrum.

### 3.3 Correlation analysis between spectral reflectance and anthocyanin concentration

**Figure 5:**
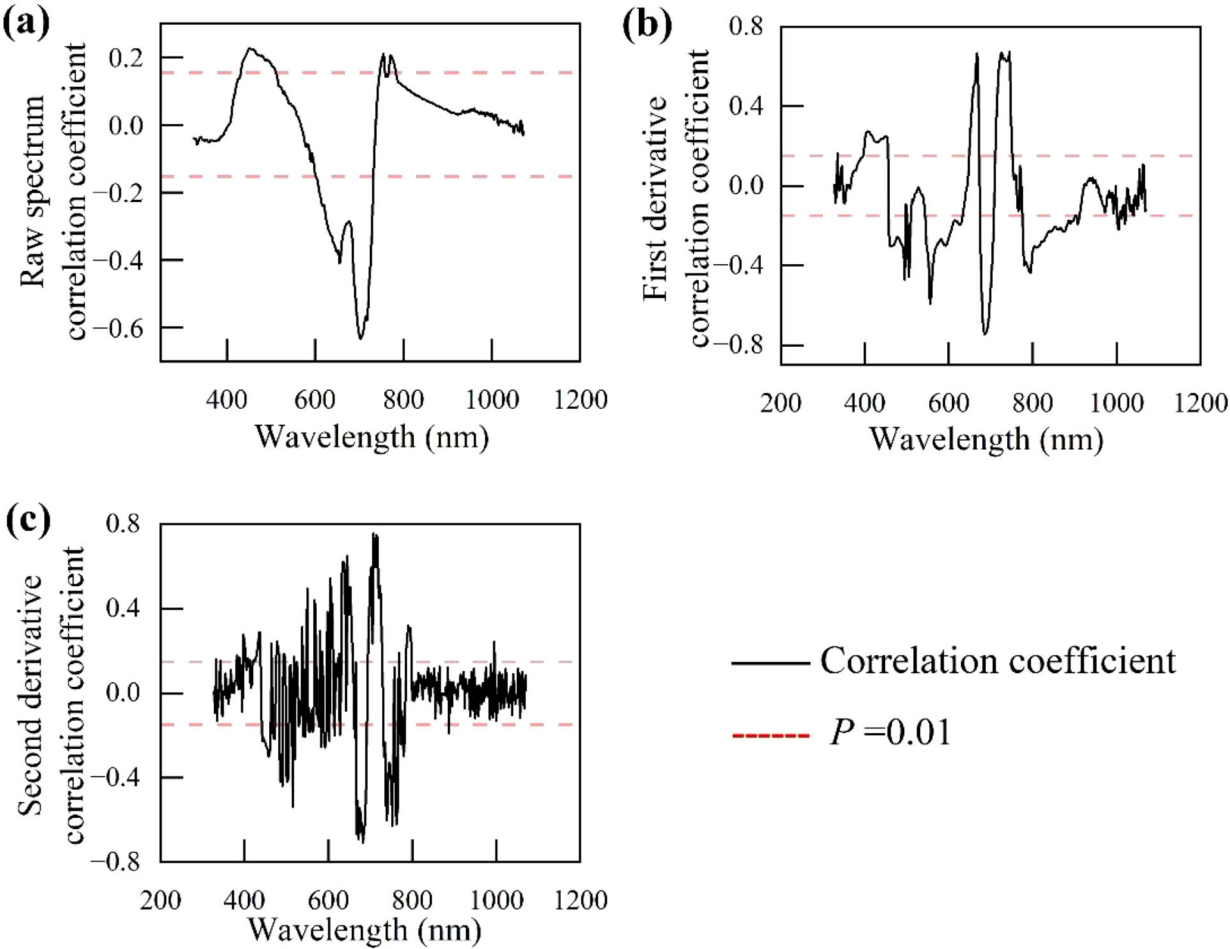
Correlation analysis between different spectral reflectance and anthocyanin concentration. **(a)** Correlation coefficient between the raw spectrum and anthocyanin concentration. **(b)** Correlation coefficient between the first-derivative spectrum and anthocyanin concentration. **(c)** Correlation coefficient between the second-derivative spectrum and anthocyanin concentration.

The first-order and second-order derivative transformations on the raw spectrum significantly enhanced the correlation between the reflectance of the transformed spectrum and anthocyanin concentration, as well as the quantity of significantly correlated bands (**Figure 5**).

There was a significantly positive correlation (*p<* 0.01) between the spectral reflectance in 420–515 nm (blue region) in the visible region and the anthocyanin concentration (**Figure 5a**). There was a significantly negative correlation (*p* < 0.01) between the spectral reflectance in the red region (600–740 nm) and anthocyanin concentration, especially at 704 nm (correlation coefficient: -0.65). Compared with the raw spectrum, the first-order and second-order derivative spectra had a larger variation of correlation coefficient and more prominent peaks (**Figures 5b, c**). A significantly negative correlation peak appeared in 680–710 nm, while positive correlation peaks appeared in 645–670 and 710–750 nm, with a maximum correlation coefficient of 0.68 (**Figure 5b**).

The curve of the second-order derivative spectra (**Figure 5c**) showed a larger variation and fluctuated more frequently, with more high-frequency noise, compared with that of the raw and first-order derivative spectra. There were multiple correlation peaks distributed in 500–720 nm, particularly at 680 nm (correlation coefficient: -0.76). In addition, some weak-correlation signals in the second-order derivative spectra appeared in the blue and near-infrared bands, but the stability and significance were not as good as those of the first derivative. Overall, the first-order derivative transformation performed the best in enhancing sensitivity and the relationship between spectrum and anthocyanin concentration.

### 3.4 Correlation analysis between different vegetation indices and anthocyanin concentration

**Figure 6:**
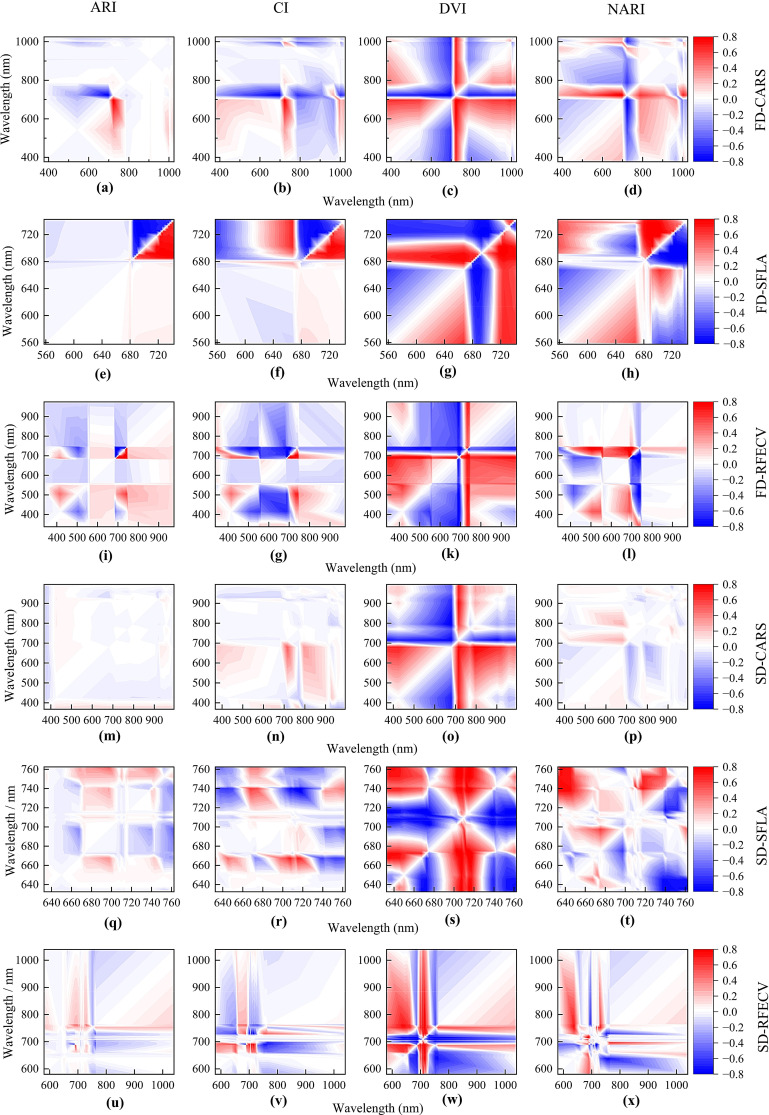
Heat map of the correlations between vegetation indices and anthocyanin concentration. The correlation results between 24 vegetation indices and anthocyanin concentration were obtained based on first-order (FD) and second-order (SD) derivative spectra, three feature extraction methods [competitive adaptive reweighted sampling (CARS), shuffled frog leaping algorithm (SFLA), recursive feature elimination with cross validation (RFECV)], and four vegetation indices [anthocyanin reflectance index (ARI), composite index (CI), difference vegetation index (DVI), and normalized anthocyanin reflectance index (NARI)]. **(a–d)** are FD-CARS-ARI, CI, DVI, and NARI, respectively. **(e–h)** are FD-SFLA-ARI, CI, DVI, and NARI, respectively. **(i–l)** are FD-RFECV-ARI, CI, DVI, and NARI, respectively. **(m–p)** are SD-CARS-ARI, CI, DVI, and NARI, respectively. **(q–t)** are SD-SFLA-ARI, CI, DVI, and NARI, respectively. **(u–x)** are SD-RFECV-ARI, CI, DVI, and NARI, respectively.

There were certain differences in the correlation of band combinations with anthocyanin concentration under different feature extraction methods (**Figure 6**). The vegetation index constructed based on the combination of spectral features in the red region (640–680 nm) and the transition zone from red edge to near-infrared region (680–760 nm) showed a high correlation with anthocyanin concentration, with correlation coefficients of 0.6-0.8. The correlation between the vegetation indices ARI and CI, constructed based on the combination of spectral features and anthocyanin concentration, was not high. The DVI and NARI constructed based on the combination of spectral features in the red and near-infrared region exhibited a high correlation with anthocyanin concentration. DVI had the highest correlation with anthocyanin concentration, followed by NARI, CI, and ARI. The vegetation indices constructed using spectral features extracted by SFLA and RFECV showed a more concentrated distribution in the red and near-infrared regions, demonstrating the advantages of these methods in extracting anthocyanin-correlated bands.

### 3.5 Optimal feature combination for constructing the vegetation index

The feature bands extracted by CARS, SFLA, and RFECV were predominantly distributed in the visible-near-infrared region (**Table 2**). Moreover, the band combinations involved in vegetation indices that exhibited higher correlations with anthocyanin concentration were primarily concentrated in the red-edge region (680–750 nm) (**Table 3**).

**Table 2:** Wavelength sets selected by different feature extraction methods.

Data type	Feature extraction method	Selected wavelengths (nm)	Number of bands
FD	CARS	377.5, 542.5, 697.5, 722.5, 787.5, 907.5, 967.5, 997.5, 1002.5, 1025	10
	SFLA	557.5, 667.5, 677.5, 682.5, 685, 687.5, 692.5, 702.5, 717.5, 722.5, 725, 737.5, 742.5	13
	RFECV	337.5, 345, 415, 505, 547.5, 557.5, 680, 692.5, 695, 702.5, 705, 732.5, 742.5, 750, 975	15
SD	CARS	372.5, 425, 680, 715, 795, 917.5, 930, 957.5	8
	SFLA	635, 637.5, 645, 667.5, 672.5, 697.5, 702.5, 707.5, 715, 737.5, 742.5, 762.5	12
	RFECV	652.5, 662.5, 687.5, 690, 707.5, 717.5, 720, 722.5, 730, 752.5, 765	11

**Table 3:** Optimal feature combination for constructing the vegetation indices.

Data type	Feature extraction method	Vegetation index	Maximum correlation coefficient	Wavelength position (i,j)/nm
FD	CARS	ARI	0.751	(722.5, 697.5)
		CI	0.737	(722.5, 697.5)
		DVI	0.787	(722.5, 697.5)
		NARI	0.796	(697.5, 722.5)
	SFLA	ARI	0.771	(725.0, 692.5)
		CI	0.764	(725.0, 702.5)
		DVI	0.783	(722.5, 702.5)
		NARI	**0.799**	**(702.5, 725.0)**
	RFECV	ARI	0.766	(732.5, 692.5)
		CI	0.742	(732.5, 702.5)
		DVI	0.758	(742.5, 695.0)
		NARI	0.782	(695.0, 732.5)
SD	CARS	ARI	0.143	(957.5, 372.5)
		CI	0.469	(715.0, 680.0)
		DVI	0.771	(795.0, 680.0)
		NARI	0.249	(680.0, 715.0)
	SFLA	ARI	0.458	(697.5, 762.5)
		CI	0.644	(707.5, 667.5)
		DVI	**0.790**	**(715.0, 672.5)**
		NARI	0.753	(635.0, 762.5)
	RFECV	ARI	0.512	(690.0, 687.5)
		CI	0.736	(707.5, 687.5)
		DVI	0.782	(707.5, 765.0)
		NARI	0.777	(690.0, 717.5)

CARS, competitive adaptive reweighted sampling; SFLA, shuffled frog leaping algorithm; RFECV, recursive feature elimination with cross-validation; FD, first-order derivative; SD, second-order derivative; ARI, anthocyanin reflectance index; CI, composite index; DVI, difference vegetation index; NARI, normalized anthocyanin reflectance index. The same below. Bold values indicate the optimal wavelength combinations and correlation coefficients used to construct vegetation indices from the first- and second-derivative spectral data.

Among the vegetation indices constructed based on the first-order derivative spectrum, the vegetation index NARI constructed using the band combination (702.5, 725) extracted by SFLA showed the highest correlation with anthocyanin concentration, with an R of 0.799, followed by NARI and DVI constructed using the band combinations (679.5, 722.5) and (722.5, 679.5) extracted by CARS, with an r of 0.796 and 0.787, respectively.

Among the vegetation indices constructed based on the second-order derivative spectrum, the vegetation index DVI constructed using SFLA (715.0, 672.5) had the highest correlation with anthocyanin concentration, with an R of 0.790, followed by the NARI constructed using RFECV extracted band combinations [(707.5, 765.0), (690.0, 717.5)], with an R of 0.782 and 0.777, respectively.

### 3.6 Modeling results

Based on first-order and second-order derivative spectral data, 12 anthocyanin concentration prediction models were constructed using three spectral feature extraction methods (CARS, SFLA, and RFECV) and two modeling methods (SVM and 1D-CNN) (**Table 4**).

**Table 4:** Performance of models constructed using different feature extraction and modeling methods.

Data type	Feature extraction method	SVM						1D-CNN					
		Training set			Validation set			Training set			Validation set		
		R^2^_c_	RMSE_c_	RPD_c_	R^2^_v_	RMSE_v_	RPD_v_	R^2^_c_	RMSE_c_	RPD_c_	R^2^_V_	RMSE_v_	RPD_v_
FD	CARS	0.944	45.012	4.212	0.830	106.288	2.423	0.693	115.828	1.804	0.626	136.113	1.635
	SFLA	0.916	59.666	3.457	**0.852**	**86.851**	**2.596**	0.670	120.076	1.740	0.640	133.744	1.664
	RFECV	0.922	62.081	3.572	0.828	79.085	2.417	0.804	94.570	2.262	**0.728**	**109.939**	**1.919**
SD	CARS	0.930	54.983	3.806	0.761	107.909	2.049	0.688	121.826	1.791	0.663	128.801	1.722
	SFLA	0.946	50.157	4.313	**0.822**	**89.329**	**2.374**	0.677	125.233	1.759	**0.674**	**126.098**	**1.752**
	RFECV	0.946	48.669	4.319	0.815	95.070	2.326	0.698	117.503	1.820	0.673	128.465	1.749

R^2^_c_, RMSE_c_, and RPD_c_ represent the coefficient of determination, root mean square error, and relative prediction deviation of the modeling set, respectively. R^2^_V_, RMSE_v_, and RPD_v_ represent the coefficient of determination, root mean square error, and relative prediction deviation of the validation set, respectively. Bold values indicate the best-performing models based on first- and second-derivative spectral data constructed using SVM and 1D-CNN.

The SVM models had better prediction performance than the 1D-CNN models. Based on the first-order derivative spectrum, the FD-SFLA-SVM model performed the best on the validation set (R^2^_V_ = 0.852, RMSE_V_ = 86.851 mg m^-2^, RPD_V_ = 2.596). Therefore, SFLA could extract bands related to anthocyanin concentration and achieve stable prediction. In contrast, the prediction performance of the FD-CARS-SVM and FD-RFECV-SVM models on the validation set was slightly lower, with an R^2^_V_ of 0.830 and 0.828, respectively. Based on the second-order derivative spectrum, the SVM models also showed high prediction accuracy, especially the SD-SFLA-SVM and SD-RFECV-SVM models (R^2^c = 0.946, RPDc > 4.3); their prediction performance on the validation set was also high (R^2^v: 0.822 and 0.815, respectively). Among the six models constructed by 1D-CNN, only FD-RFECV-1D-CNN had high prediction accuracy (R^2^_V_ = 0.804, RMSE_V_ = 94.570 mg m^-2^, RPD_V_ = 2.262). Although the second-order derivative transformation could better highlight spectral details, the prediction accuracy on the validation set was not significantly better than that of the first-order derivative transformation.

The SVM models showed high prediction performance, and their R^2^_V_ were between 0.76 and 0.85, with an RPD_V_ greater than 2. The R^2^_V_ of the 1D-CNN models were generally between 0.63 and 0.73, with an RPD_V_ smaller than 2. Therefore, under the conditions in this study (i.e., sample size and spectral features), SVM was more adaptable to feature inputs, achieving effective modeling. The SVM modeling method, combined with first-order derivative transformation and SFLA feature extraction, was the optimal solution for anthocyanin concentration prediction.

**Figure 7:**
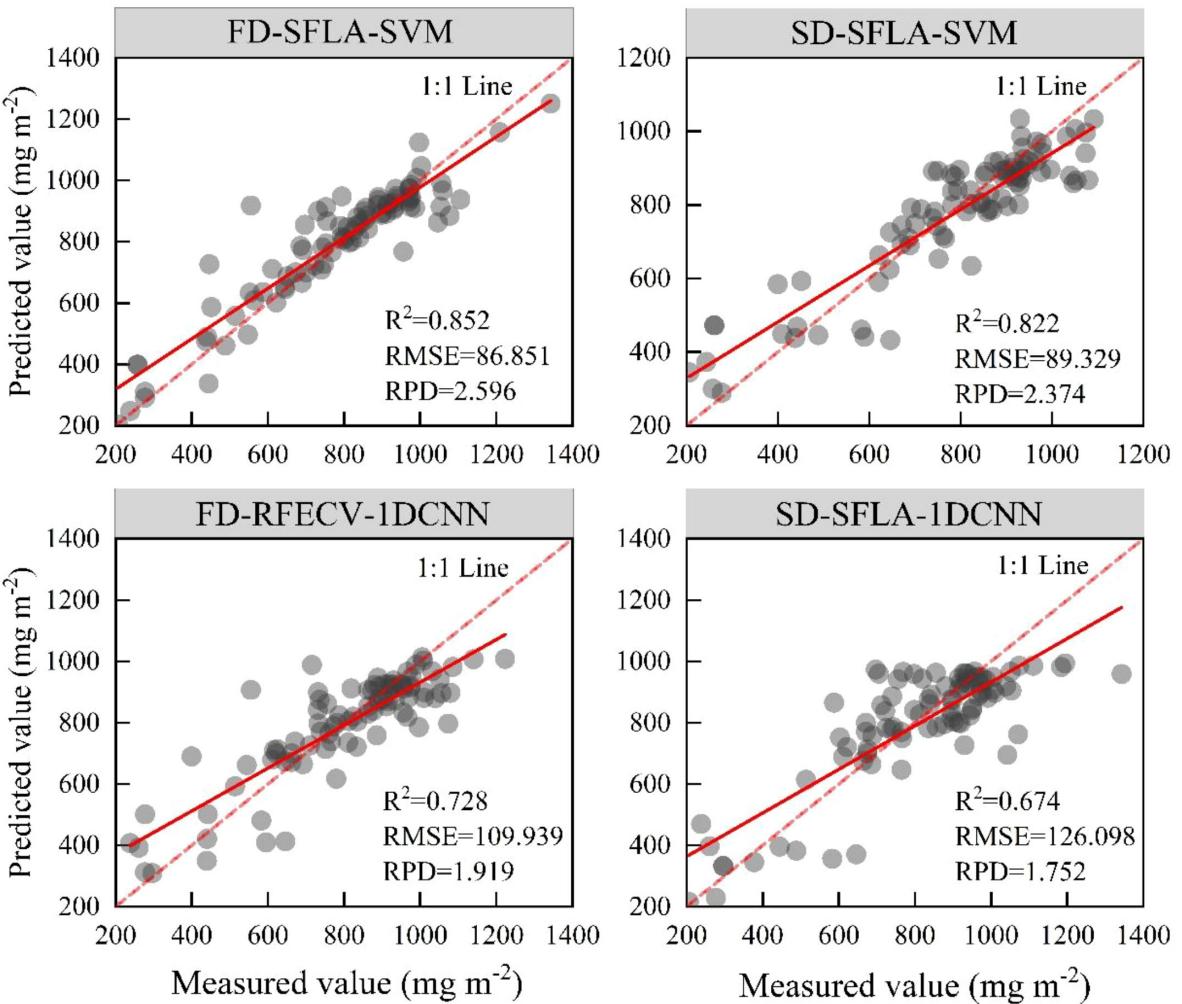
Verification of the optimal model for the prediction of anthocyanin concentration in *P. pruinosa* leaves.

The comparison of the performance (validation set) of the four optimal SVM and 1D-CNN models based on first-order and second-order derivative spectral data (**Figure 7**) demonstrated that SVM models consistently outperformed 1D-CNN models. The FD-SFLA-SVM model performed the best, with high prediction accuracy and stability. The SD-SFLA-SVM model also had high accuracy, but its prediction performance on some high-value samples was low.

## 4 Discussion

### 4.1 Spectral preprocessing enhanced the correlation between leaf spectrum and anthocyanin concentration

Spectral preprocessing can highlight target information, reduce noise interference, and enhance model accuracy and stability. Xiao et al. (2022) compared the performance of seven spectral preprocessing methods in the prediction of chlorophyll content in cotton leaves, finding that first-order derivative (FD) and standard normal variate (SNV) were the best preprocessing methods. Combined with deep learning, they provided new possibilities for evaluating the nutritional status of cotton in the field. Zhang et al. (2025) found that SG smoothing and fractional order differentiation (FOD) highlighted the spectral features of cotton field soil and cotton canopy, providing technical support for monitoring soil salinization in cotton fields using hyperspectral imaging. The soil salinity prediction model constructed using the 0.7th-order FOD combined with the bootstrap soft shrinkage (BOSS) algorithm showed the best performance, with an R^2^ of 0.92. Jiang et al. (2025) comparatively evaluated the effects of the preprocessing methods Savitzky-Golay smoothing, max normalization, and first-order derivative on the performance of machine learning models in predicting citrus soluble solids content and titratable acidity, demonstrating the importance of spectral preprocessing.

This study performed 2.5 nm-interval resampling and Savitzky-Golay smoothing on spectral data, followed by first-order and second-order derivative transformations. The preprocessed spectra showed significant enhancement in relation to anthocyanin concentration. This may be because resampling enhances the wavelength resolution of the resampled spectrum, and the first-order and second-order derivative preprocessing highlight spectral features, improving data quality and usability (Omia et al., 2023). In the correlation curves between the reflectance of first-order and second-order derivative spectra and anthocyanin concentration in this study, first-order and second-order derivative transformations significantly highlighted the anthocyanin-related bands, and the curves fluctuated more frequently, presenting more significant correlation peaks compared with the raw spectrum. This is similar to the prediction of anthocyanin concentration using near-infrared spectroscopy by Huang et al. (2014), where both visible and near-infrared bands are highly related to anthocyanin concentration.

### 4.2 Spectral reflectance in different bands responded differently to anthocyanin concentration

With the variation of anthocyanin concentration, the variation of the spectral reflectance of *P. pruinosa* leaves in 410–700 nm is similar to that of the reflectance of anthocyanin concentration in *Zea mays* var. *purpurea* predicted using hyperspectral data (Yang et al., 2022). This indicates that the pigment-dominated spectral response has a certain universality. In the visible region, the strong absorption of chlorophyll can reduce the spectral reflectance of the canopy, and environmental stress, plant structure, and other factors can also affect the spectral reflectance (Sahoo et al., 2025; Knipling, 1970). Under drought stress, the mesophyll cells become loosely arranged, leading to weakened scattering effects inside the leaves and spectral reflectance changes. Anthocyanins accumulate in large quantities under drought stress and slow down the plant aging process through photoprotection (Li and Ahammed, 2023a). Therefore, the significant differences in some bands’ reflectance under different anthocyanin concentrations in this study are likely due to the varying degrees of drought experienced by *P. pruinosa* under different groundwater depths and the weakened absorption of chlorophyll.

In the blue region, leaves undergo drought-induced aging, and chlorophyll begins to degrade, leading to a decrease in light absorption. At the same time, anthocyanins gradually accumulate. Although anthocyanins also undergo absorption in the blue region, the increase in reflectance caused by chlorophyll degradation dominates, resulting in an increase in spectral reflectance with increasing anthocyanin concentration. Studies have shown that under drought stress, the accumulation of anthocyanins mainly functions in light shielding and anti-photooxidation, rather than predominantly absorbing blue light (Li and Ahammed, 2023b). Therefore, the increase in blue-region reflectance is mainly attributed to the reduced blue light absorption capacity caused by chlorophyll degradation. When the anthocyanin concentration reached the maximum range of 1,000-1,200 mg m^-2^, the spectral reflectance was 0.18, which was the maximum value in the blue region.

In the red region, the decrease in reflectance involves complex energy regulation mechanisms. Drought stress activates the non-photochemical quenching (NPQ), which dissipates excess excitation energy as heat, thereby protecting photosystem II (Demmig-Adams et al., 2012). Meanwhile, the accumulation of anthocyanins in the epidermal layer enhances the light-shielding effect, reducing the risk of photoinhibition (Gould et al., 2010). This effect exceeds the increase in reflectance caused by chlorophyll degradation. Thus, in this study, reflectance was negatively correlated with anthocyanin concentration and remained at a low level (dropping to 0.14 at 677 nm).

In addition, drought can cause water loss and shrinking of leaf mesophyll cells and reduce intercellular space, disrupting internal scattering of leaves and thus altering spectral reflectance characteristics (Sukhova et al., 2025). This leaf structural adjustment, together with pigment regulation, constitutes the adaptation strategy of *P. pruinosa* to the changes in groundwater level. Therefore, the spectral differences under different anthocyanin concentrations not only reflect changes in pigments, but also characterize their comprehensive physiological response to drought.

### 4.3 Vegetation indices enhanced the response of spectral reflectance to anthocyanin concentration

The vegetation index can predict the physiological and biochemical components of plants (Zeng et al., 2022), eliminate the influence of background noise, and reflect changes in the components (Hallik et al., 2017). Spectral feature extraction can reduce data redundancy; the models constructed based on spectral features-based vegetation index have high stability (Zhang et al., 2024). This study used CARS, SFLA, and RFECV for feature extraction. It was found that SFLA and RFECV had significant advantages in extracting anthocyanin concentration-related bands. These spectral features can reflect both the optical property changes caused by anthocyanin accumulation (red edge region) and the stress-induced leaf structure changes (near-infrared region). This study constructed vegetation indices based on the extracted feature bands to characterize the dynamic changes in anthocyanin concentration under drought stress. Compared with the traditional vegetation indices constructed using the full band, the correlations between the feature bands-based vegetation indices and the target variable are higher. This enhances the prediction stability and accuracy. This is the difference between the proposed approach and the existing vegetation index-based studies. These indices are closely related to anthocyanin concentration in *P. pruinosa* leaves, with a correlation coefficient of 0.6-0.8. Gamon and Surfus (1999) constructed a red/green index (R/G) that can be used for non-destructive prediction of anthocyanin and chlorophyll concentrations, based on the principle of anthocyanin absorption at 550 nm in the green region and chlorophyll absorption in the red and green regions. Lopes et al. (2017) explored the feasibility of using modified vegetation indices, DSI (difference spectral index), RSI (ratio spectral index), and NDVI (normalized difference spectral index), to predict the concentration of carotenoids and anthocyanins in lettuce. Viña and Gitelson (2010) successfully evaluated the relation of four broadband vegetation indices to anthocyanin concentration using the green-region reflectance of five plant species. In addition, scholars (Gitelson et al., 2009; Ritchie et al., 2010) have also constructed the Red/Green Index (RG), Anthocyanin concentration Index (ACI), and Modified Anthocyanin concentration Index (MACI) for anthocyanin prediction by selecting highly-related bands in the visible and near-infrared regions. This study found that there was a high correlation between vegetation indices in the red region (640–680 nm) and the transition zone from red edge to near-infrared region (680–760 nm) and anthocyanin concentration in *P. pruinosa* leaves. This is similar to the research results of Kross et al. (2015). They used resampled bands to construct vegetation indices, which not only enhanced the efficiency of spectral data utilization but also enhanced the specificity and sensitivity of soybean chlorophyll content monitoring. Overall, vegetation indices are of great significance for predicting anthocyanin concentration in *P. pruinosa* leaves, and the vegetation indices have varying effects under different conditions.

### 4.4 SVM modeling strategy was better than 1D-CNN in leaf anthocyanin concentration prediction

Traditionally, the anthocyanin concentration in plant leaves is detected mainly using spectrophotometry based on the spectral absorption characteristics of plants. This method is time-consuming and laborious, and damages the leaf tissues, limiting its widespread application. In recent years, the combination of machine learning algorithms and hyperspectral technology has provided a new solution for rapid, non-destructive, and real-time monitoring of plant anthocyanins. Liu et al. (2019) used principal component regression (PCR), partial least squares regression (PLSR), and back propagation neural network to construct models for predicting anthocyanin concentration in cherry leaves based on reflectance data (450–600 nm). Dai et al. (2023) constructed a variety identification model based on anthocyanin concentration in fresh leaves of Zijuan tea using principal component analysis combined with PLSR and support vector regression (SVM), yielding an identification accuracy of over 90%. The addition of biophysical variables (i.e., water shortage) can improve model performance (Sandoval et al., 2025). This study used two machine learning methods, SVM and 1D-CNN, to predict the leaf anthocyanin concentration of *P. pruinosa* in different groundwater depth conditions in arid areas, showing different prediction accuracy. Based on the preprocessing of spectral data, the SVM and 1D-CNN prediction models constructed by using the vegetation indices constructed based on the spectral features extracted by CARS, SFLA, and RFECV mostly performed well, with FD-SFLA-SVM, SD-SFLA-SVM, FD-RFECV-1D-CNN, and SD-SFLA-1D-CNN models showing higher accuracy.

In this study, the SVM model generally outperformed the 1D-CNN model in predicting anthocyanin concentration in *P. pruinosa* leaves. This difference can be explained by the dataset structure, feature representation, and model complexity. Firstly, SVM, based on the principle of structural risk minimization, can handle nonlinear regression tasks with limited samples, offering distinct advantages (Mountrakis et al., 2011). Its advantages are fully reflected in this study with a moderate sample size and a medium-dimensional feature space (n = 303, *p* = 40). In contrast, the 1D-CNN struggles to support parameter learning for a deep network with only 303 samples in this study, making it prone to overfitting. Secondly, 1D-CNNs excel at leveraging the continuity of spectral data. However, because vegetation indices were constructed based on discrete feature bands, the continuity of vegetation indices has been disrupted, making it difficult for the 1D convolutional structure to fully utilize its advantage in extracting local spectral features. This is consistent with the study conclusions of Hu et al. (2015). Thirdly, compared with the complex structure and large number of parameters of 1D-CNN, SVM, with its convex optimization and streamlined hyperparameters, demonstrates better generalizability and stability on medium-sized datasets. Overall, under moderate sample sizes and vegetation index-based modeling, SVM exhibits higher stability and generalizability than 1D-CNN (Camps-Valls et al., 2013). The model constructed using SVM, namely FD-SFLA-SVM model, performed the best (R^2^_V_ = 0.852, RMSE_V_ = 86.851 mg m^-2^, RPD_V_ = 2.596).

## 5 Conclusion

The reflectance spectrum varies with the physiological parameters in plant leaves, which necessitates the timely monitoring of the physiological parameters in the assessment of plant growth status. This study preprocessed and resampled spectral data and then performed first-order and second-order derivative transformations on the spectral data. This enhanced the accuracy of spectral data analysis and interpretation, highlighted anthocyanin concentration-related bands, and enhanced data availability. Three methods, CARS, SFLA, and RFECV, were used to extract the spectral features of the raw spectrum. The spectral features were mainly concentrated in the red region of 640–760 nm. SFLA and RFECV had significant advantages in extracting anthocyanin concentration-related bands. The vegetation index NARI constructed using the spectral features extracted from the first-order derivative spectral data by SFLA had the highest correlation with anthocyanin concentration, with an R value of 0.799. The vegetation index DVI had the highest correlation with anthocyanin concentration, followed by NARI, CI, and ARI. The bands with high correlation were concentrated in the red region (640–680 nm) and the transition zone from red edge to near-infrared region (680–760 nm). A total of 12 anthocyanin concentration prediction models were constructed using SVM and 1D-CNN. The six models constructed by SVM were overall superior to the corresponding six 1D-CNN models. Among them, the FD-SFLA-SVM model performed the best (R^2^_V_ = 0.852, RMSE_V_ = 86.851 mg m^-2^, RPD_V_ = 2.596). In future research, it is necessary to further address how to better utilize sample data to make the model applicable to different stresses and enhance the accuracy and universality of the model.

## Data Availability

The raw data supporting the conclusions of this article will be made available by the authors, without undue reservation.
